# Surgical Repair of post-infarction ventricular septal rupture: Determinants of operative mortality and survival outcome analysis

**DOI:** 10.12669/pjms.341.13906

**Published:** 2018

**Authors:** Muhammad Yasir Khan, Tariq Waqar, Perisa Gul Qaisrani, Adnan Zafar Khan, Muhammad Shahrukh Khan, Haider Zaman, Anjum Jalal

**Affiliations:** 1 Dr. Muhammad Yasir Khan, MCPS, FCPS(G.S), FCPS(C.S), MRCS. Department of Cardiac Surgery, Ch. Pervaiz Elahi Institute of Cardiology, Multan, Pakistan; 2 Dr. Tariq Waqar, FCPS, FRCS Department of Cardiac Surgery, Ch. Pervaiz Elahi Institute of Cardiology, Multan, Pakistan; 3 Dr. Perisa Gul Qaisrani, MBBS. Department of Medicine, Ibn-e-Sina Hospital Multan, Pakistan; 4 Dr. Adnan Zafar Khan, MBBS, MSc Health economics Health Department Punjab Govt, Lahore, Pakistan; 5 Muhammad Shahrukh Khan, Quaid-e-Azam Medical College, Bahawalpur, Pakistan; 6 Prof. Dr. Haider Zaman, FCPS, FRCS Cth. Department of Cardiac Surgery, Ch. Pervaiz Elahi Institute of Cardiology, Multan, Pakistan; 7 Prof, Dr. Anjum Jalal, FCPS, FRCS Cth. Chief Executive and Head of Cardiac Surgery Department, Faisalabad Institute of Cardiology, Faisalabad, Pakistan

**Keywords:** Ventricular septal rupture, Myocardial infarction, Coronary artery bypass, Cardiogenic shock

## Abstract

**Background and Objective:**

Ventricular septal rupture (VSR) is one of the fatal complications of myocardial infarction (MI). Surgery provides the maximum survival benefit. Our objective was to investigate the risk factors of surgical mortality and to do the survival analysis in the past six years at our hospital.

**Methods:**

All the patients operated at CPE Institute of Cardiology Multan Pakistan, between 2009 and 2015 for repair of post MI VSR were analysed retrospectively for demographics, comorbidities, operative and post operative outcomes. The primary outcome was 30 days mortality. The follow up was done till April 2017 and the follow up data was obtained from hospital records and by telephoning the patients. SPSS was used for statistical analysis. P value < 0.05 was considered significant.

**Results:**

A total of 31 patients were operated for VSR repair with a mean age of 57.19±7.73 years. Eighteen patients also had a concomitant coronary artery bypass grafting (CABG). The operative mortality in this series was 25.8% Univariate analysis showed that pre-operative ejection fraction (E.F) (p value 0.010) and cardiogenic shock (p value 0.031) were a significant risk factors for operative mortality while on logistic regression analysis only the cardiogenic shock was found to be an independent risk factor for operative mortality with the odds ratio of 2.17. Low ejection fraction only acted as a confounding variable. The mean survival at six years was 34 months with a survival rate of 28.6%. The additional CABG did not confer any survival benefit.

**Conclusion:**

The patients in cardiogenic shock pre-operatively have a high operative mortality. Low ejection fraction (E.F) acts as a confounding factor. Concomitant CABG does not confer any survival benefit.

## INTRODUCTION

Rupture of the interventricular septum is a fatal complication of acute myocardial infarction. It presents 2-8 days after an infarction and unless surgically treated, most of these patients die of severe congestive cardiac failure within hours to days.^1^ Prior to the era of widespread reperfusion therapy the reported incidence of this complication was 1% to 3%^2^ which has declined to 0.3% following the advent of thrombolysis.^3^ According to the Assessment of Pexelizumab in Acute Myocardial Infarction (APEX AMI) trial, VSR typically occurs much earlier ranging from 8-24 hours after AMI and the, rates of mechanical complications like VSR were lower with primary PCI than those previously reported after fibrinolytic therapy.^4^ Nonoperative treatments for post-infarction ventricular septal defect (VSD) have poor results. Historical reviews have shown that 50% of patients died within one week, 80% within four weeks, and less than 10% survived beyond one year.^4^ According to the GUSTO-I trial the 30 day mortality was 47% of the patients who had surgical repair of VSR as against 94% for those patients who did not undergo surgery.^3^

Surgical repair of post-infarction VSD is the definitive treatment of choice.^5^ Location of the VSD varies depending upon the coronary artery involved. It can be anterior, apical and posterior. A posterior VSD in particular may be accompanied by mitral valve regurgitation secondary to papillary muscle infarction or ischemia. The disease pattern differs between the Indian subcontinent and the Western populations so all the determinants may not apply to the Asian populations.

This retrospective single centre study aimed to investigate the risk factors of operative mortality associated with surgical repair of post-infarction VSR and the survival analysis at a tertiary referral centre for last six years.

## METHODS

After approval from the institutional ethical committee, data of all the patients operated between 2009 and 2015, at CPE Institute of Cardiology Multan Pakistan, for post MI VSD was retrieved from cardiac surgery database of the hospital and was retrospectively analyzed. The patients who developed VSD after MI diagnosed on echocardiography were included. The patients who presented with this condition and had already developed multi-organ failure or died before being offered surgery were excluded. Angiography was performed in all the patients who were later on operated. Thirty One primary post-infarction VSD operations were performed in our unit at Ch Pervaiz Elahi Institute of Cardiology Multan in this six years period. The patients who had concomitant CABG along with VSR were also included. The demographics, clinical presentation, comorbidities, echocardiographic data e.g ejection fraction, location of the VSD, use of IABP, surgical procedures and outcomes were reviewed.

All the patients were operated on standard cardiopulmonary bypass with infarct exclusion technique while in some patients aortic cross clamp was not used and surgery was performed on cardiopulmonary bypass with beating heart and no cross clamp applied. The left ventricle was opened through the infarct either anteriorly or posteriorly in cases of anterior or posterior infarcts respectively. The margins identified and VSD was repaired using a dacron patch with 4/0 prolene in an interrupted manner. The left ventricle was then repaired with 3/0 prolene over a Teflon felt to buttress the repair. Concomitant CABG was not performed on all the patients but where feasible according to the narrowing of the vessels. The patients who were on inotropes pre-operatively and were operated on within 24 hours were labelled as emergent. Acute kidney injury (AKI) was labelled according to KIDCO classification as 1.5 times increase of serum creatinine than the baseline.

The primary outcome variable was in hospital mortality labeled as death from any cause within 30 days of operation. The secondary variables were presence of cardiogenic shock, bypass time, cross clamp time, ICU stay and other comorbidities like re-exploration for bleeding and residual VSD. The follow up data was obtained from hospital records and by telephoning the patients. The follow up was done till April 2017.

Frequencies are presented as number and percentage for the quantitative variables presented as mean±SD or median and range. Student t test and chi sq test were used for statistical comparisons. Univariate analysis was performed on all the variables. Subsequently a multivariate analysis was carried out on these variables by constructing a logistic regression model to identify the independent risk factors of post-operative mortality. Kaplan Meier analysis was used to estimate the survival function. Values of p<0.05 were considered statistically significant. All the statistical analysis was done using SPSS 20.

## RESULTS

**Fig. 1:**
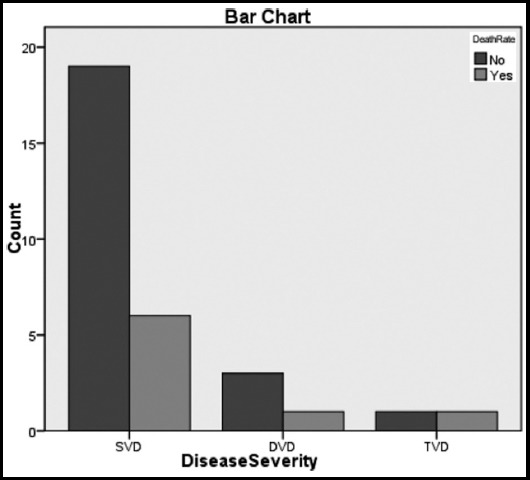
Disease Severity among survivors and non-survivors.

**Fig. 2:**
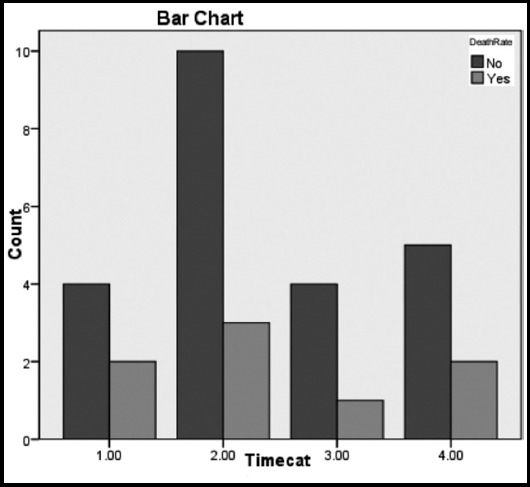
Time scale from diagnosis to operation among survivors and non survivors. *Legend:* 1= 1-3 days 2= 4-7 days 3= 8-14 days 4= > 14 days (p= 0.97)

Between the years 2009 and 2015 total of 31 patients were operated for VSR repair with or without concomitant CABG. Mean age of the patients was 57.19±7.73 years. Out of these 31 patients 21 (67.7%) were male while 10 (32.3%) were female. Thirteen (42%) patients were smokers and diabetic while only 8(26%) patients had hypertension. The mean body surface area of all the patients was 24.8±4.56. Majority of the patients 16 (52%) were in NYHA class III pre-operatively with a mean ejection fraction of 38%±7.61. Angiography was performed in all the patients. About 80.6% (25) patients had single vessel disease while 13% (4) had double vessel and 6.5%(2) patients had three vessel disease. Fig.1 shows the disease severity among the survivors and the non survivors. Concomitant CABG was performed in 18 (58.1%) patients while 13 (42%) patients had only VSR repair. The mean time from the diagnosis of VSR and surgical repair was 9.35±7.88 days. Fig.2 shows time scale from diagnosis to operation among survivors and non survivors. Emergency operation within 24 hours of presentation was performed in four patients, 2 (50%) of them died but no statistically significant difference was observed between survivors and non survivors. Intra-aortic balloon pump (IABP) was used in 13 patients and out of these patients 7 (22.6%) had it passed pre-operatively. Seven patients presented with cardiogenic shock and out of these four died. The kidneys of 4 (17.4%) patients had a hit pre-operatively, were in acute kidney injury (AKI) but none of them died. Similarly no patient required renal replacement therapy post-operatively. In terms of location, the apical VSR's were more in number as compared to the posteriorly located ones, 84%(26) vs 16%(5) respectively. The 30 day or operative mortality in this series was 25.8% (8 patients). Out of 23 (74.1%) patients who survived, a residual VSD was seen in 3(9.7%) patients on echocardiography post-operatively. None of these patients was unstable enough to warrant any surgical intervention for residual VSD. Re-exploration for bleeding was done in only one patient who later on recovered and was sent home in a satisfactory condition. As all the patients were operated using standard cardiopulmonary bypass but in six patients no crossclamp was used and they were operated on beating heart without aortic crossclamp. Among these six patients only one died. On statistical analysis there was no significant difference in mortality among patients with aortic cross clamp vs those who had no cross clamp applied (p value 0.57). Comparison of the pre-operative, intra-operative and post-operative was performed among the survivors and the non survivors and is shown in Table-I. Univariate analysis of the comparison revealed that pre-operative ejection fraction (p value 0.010) and cardiogenic shock (p value 0.031) were a significant risk factors for operative mortality. When both of these risk factors were analyzed using logistic regression, it was found out that only the cardiogenic shock was the independent risk factor for operative mortality with the odds ratio of 2.17. The low ejection fraction added to the operative mortality and acted as a confounding variable. Our results show that the patients in cardiogenic shock with low ejection fraction are independent risk factors for operative mortality. This is explained in Table-II.

**Table-I:** Comparison of pre-operative, intra-operative and post-operative variables. N=31

Variables	Alive patients n=23	Patients not alive n=8	P-value
*Pre-operative*			
Smoking	11	2	0.26
Hypertension	6	2	0.95
Diabetes	10	3	0.78
IABP pre-op	3	4	0.055
Intra-op	6	
NYHA class	3.0±0.98	3.2±0.41	0.2
Ejection fraction	39±8.34	34.38±3.2	0.010
Creatinine (mg/dl)	1.53±1.29	1.15±0.07	0.37
AKI pre-op	4	0	0.21
Cardiogenic shock	3	4	0.031
Time to operation (days)	9.5±8.2	8.9±7.38	0.97
Emergency Operation	2	2	0.24
*Intra-operative*			
Concomitant CABG	12	6	0.26
No of grafts	0.9±0.95	1.1±1.35	0.34
CPB time (min)	117±47.36 (103 median)	129±63.0 (123 median)	0.89
Cross clamp time (min)	57.74±40.68 (58 median)	73.2±50.94	0.50
Position of VSD Apical	20	6	0.43
Posterior	3	2	
*Post-operative*			
ICU stay (hours)	75.13±7.52 (48 median)	46.5±1.35 (48 median)	0.21
Ventilation time (hours)	37.95±8.1 (16 median)	10±.5 (10 median)	0.52
Inotropes duration (hours)	48.47±3.71	16.5±1.06	0.06
Hospital stay (days)	11±1.34		
Chest drainage (ml)	854±6.21	740±4.38	0.78

**Table-II:** Multivariate analysis using Log regression.

Variable	Odds Ratio	95% confidence interval	p-value
Ejection fraction	-0.134	0.732-1.045	0.15
Cardiogenic shock	2.177	1.11-69.89	0.039

**Fig. 3:**
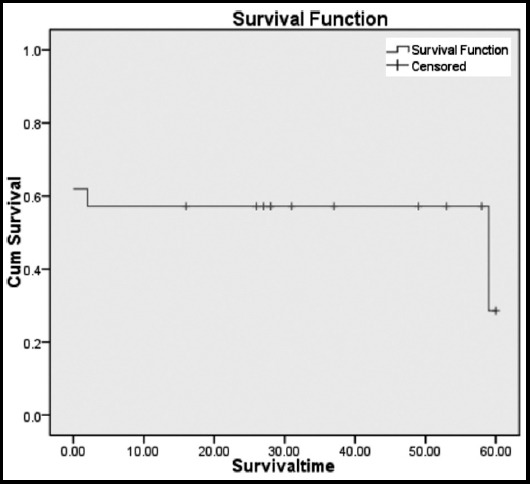
Kaplan-Meier survival analysis.

At the time of discharge from the hospital 23(74%) patients were alive. One of the patients was readmitted with multi-organ system failure two months after the discharge and died later on. At the end of six years 10 patients had died with 11 patients lost to follow up. The mean survival at six years was 34.095 months with survival rate of 28.6%. This is clearly shown in Kaplan-Meier survival analysis in Table-III and the Fig.3 explains the survival plot. The addition of coronary revascularization did not produce any survival benefit.

**Table-III:** Kaplan-Meier survival analysis.

No of cases	No. Censored	No. of events	Median survival (months)	Mean survival in months (95%CI)	%age survival
31	11	10	59	34.095 (21.5-46.6)	28.6%

## DISCUSSION

VSR complicating acute MI is still one of the most challenging morbidity which cardiac surgeons have to face today. Although with the introduction of reperfusion therapies, the standard of practice in the treatment of AMI, the incidence of VSR has decreased to 0.17-0.31% as compared to the pre-thrombolytic era when it was 1-3%, but in the previous two decades, the rate of VSR complicating AMI has not changed and the mortality associated with VSR has remained high and relatively constant.^6^

The 30 days operative mortality from our study results was 25.8% which is much lower than reported in some other series where mortality rate of 34% to 37%^7^-^10^ has been found. Likewise lower mortality rates have also been reported ranging from 19% to 25%,^11^,^12^ on comparison of survivors with non survivors after doing univariate analysis lower ejection fraction and the presence of cardiogenic shock were identified as predictors of operative mortality. It was also demonstrated by Pang PY et al.^13^ and Haung et al.^14^ who demonstrated that preserved LVEF has a positive impact on early survival. The cardiogenic shock is partly due to decreased contractility of the infarcted segment and the presence of left to right shunt. In other studies the duration of aortic cross clamp and increased cardiopulmonary bypass^15^ time have been identified as predictors of early mortality but no such association was found in this series. Moreover the mortality rate was no different in our patients who were operated on beating heart with no aortic cross clamp.

From the results of some studies the time interval between the diagnosis of VSR and surgical repair is of utmost importance. The longer the interval before surgery, the better the survival is,^16^,^17^ delayed repair lends time for fibrosis and myocardial scar tissue formation around the VSD and thus allows for a better and long lasting repair with less chances of residual VSD's post operatively, due to this reason many centres use mechanical circulatory support in the form of IABP, Impella, Tandem Heart and ECMO pre-operatively to hemodynamically stabilize the patients and delay the surgery till the second week. We used only IABP in seven patients pre-operatively in six patients intra-operatively. We followed the ACC/AHA guidelines and inserted IABP in all those patients who were in cardiogenic shock.^18^ In our series the mean time from diagnosis to surgery of VSR was 9.5 days and 8.9 days among survivors and non survivors respectively with no statistically significant difference between the survival groups (p 0.97). Some studies have shown that IABP improved survival until a surgical repair of VSR was performed.^19^ We also observed that survival was nearly significant with the use of IABP (p value 0.055) but on the contrary according to a retrospective re-view of 2,876 patients from The Society of Thoracic Surgeons (STS) Adult Cardiac Surgery Database (ACSD), the use of IABP both preoperative and intraoperative had significantly higher mortality outcomes.^20^ Moreover early repair occurring less than seven days post-MI had much greater mortality than delayed repair occurring more than seven days post-MI (54.1% vs 18.4%, p<0.01).^20^

It has been observed that AKI requiring dialysis post operatively is an independent risk factor of mortality.^13^ In our series none of the patients received renal replacement therapy post operatively. Similarly in other studies right ventricular dysfunction has been found to have a negative impact on early and late survival.^21^ None of our patients had RV dysfunction as well.

Among our study subjects, we performed concomitant CABG in 18(58.1%) of the patients, while different studies have shown different results in terms of survival advantage of revascularization in addition to VSR repair. According to Lundblad et al.^22^ concomitant CABG reduces both early and late mortality. Similarly Perotta et al.^23^ reported improved mortality rates from 26.3% to 21.2% when CABG was performed concomitantly. On the other hand some series have only reported mid to long term survival benefit with CABG and no significant impact on early mortality.^24^ In our study we did not find any statistically significant early or long term survival benefit of concomitant CABG with VSR repair.

Percutaneous device closure of the post MI VSR is being increasingly used these days especially in those patients who are a high surgical risk or as a temporary measure as a bridge to surgery.^25^ It can also be used in patients with hemodynamically significant residual shunts. This method is not suitable for large (>15mm) VSR's who should immediately undergo surgical correction. Thirty day mortality rates range from 28% - 65%^25^,^26^ while patients in cardiogenic shock have much higher mortality 88% vs 38%, with this modality of treatment.^25^

Ventricular assist devices are a useful adjunct in the setting of uni-ventricular and bi-ventricular failure either as a bridge to surgery or post-operatively. It allows better peripheral organ perfusion and recovery of the hibernating myocardium before the VSR repair. A similar bridge to surgery approach has been reported by La Torre et al using an Impella pump in patients with posterior VSR's.^27^

In this series lower ejection fraction and the presence of cardiogenic shock were found to be independent risk factors of early mortality. On multivariate analysis using log regression only cardiogenic shock was found to be the independent risk factor while lower ejection fraction only acted as a confounding variable. It means that patients in cardiogenic shock with lower ejection fraction had more chances of dying. Our results are supported by the SHOCK (should we emergently revascularise occluded coronaries for cardiogenic shock?) trial which showed that the hospital mortality for surgical repair of VSR with patients in cardiogenic shock was 81%.^28^

### Limitations of the study

Our study subjects consist of small retrospective data from our institution so for some of the variables statistical tests are not statistically significant.

## CONCLUSION

From the results of our study it is evident that preoperative evidence of low ejection fraction especially in patients of cardiogenic shock have significantly higher surgical mortality, Among unstable patients the early insertion of IABP may improve the outcome post-operatively. Though concomitant CABG had no impact on early mortality or late survival but should be performed in patients with multi-vessel disease as long as it can be performed safely.

### Authors' Contribution

**MYK** conceived and designed the study and collected in hospital data.

**TW and PGQ** did manuscript writing.

**AZK and MSK** collected the six years follow up data and did statistical analysis.

**HZ and AJ** did review and final approval of manuscript.
